# Linear Deterministic Accumulator Models of Simple Choice

**DOI:** 10.3389/fpsyg.2012.00292

**Published:** 2012-08-23

**Authors:** Andrew Heathcote, Jonathon Love

**Affiliations:** ^1^School of Psychology, The University of NewcastleCallaghan, NSW, Australia

**Keywords:** evidence accumulation, mathematical modeling, response time, linear ballistic accumulator, lexical-decision task

## Abstract

We examine theories of simple choice as a race among evidence accumulation processes. We focus on the class of deterministic race models, which assume that the effects of fluctuations in the parameters of the accumulation processes between-choice trials (*between-choice noise*) dominate the effects of fluctuations occurring while making a choice (*within-choice noise*) in behavioral data (i.e., response times and choices). The latter deterministic approximation, when combined with the assumption that accumulation is linear, leads to a class of models that can be readily applied to simple-choice behavior because they are computationally tractable. We develop a new and mathematically simple exemplar within the class of linear deterministic models, the Lognormal race (LNR). We then examine how the LNR, and another widely applied linear deterministic model, Brown and Heathcote’s (2008) LBA, account for a range of benchmark simple-choice effects in lexical-decision task data reported by Wagenmakers et al. (2008). Our results indicate that the LNR provides an accurate description of this data. Although the LBA model provides a slightly better account, both models support similar psychological conclusions.

## Introduction

Humans and other organisms often have to respond to stimuli under time pressure that requires them to make choices in a few seconds or less. In contrast to complex choices requiring an extended period of deliberation during which a long series of cognitive operations are completed, rapid choices are usually assumed to have a simple cognitive architecture consisting of three stages: stimulus encoding, response selection, and response execution. Response selection in simple choice is almost universally modeled by evidence accumulation, that is, by a process that accumulates evidence until the amount favoring one of the choices is sufficient to exceed an evidence boundary. Evidence accumulation has the disadvantage that it becomes increasingly time consuming when the evidence boundary is high. However, increasing response caution by increasing the boundary is also assumed to have utility because it ameliorates the effects of various types of noise that can cause choice errors. This assumption, and the task of providing a quantitative account of the relationship between speed and accuracy (*speed-accuracy trade-off*), has been pivotal for the development of models of simple choice.

Since the earliest proposals (e.g., Stone, 1960), it has usually been assumed that fluctuations in evidence occurring during the accumulation process (i.e., *within-choice noise*) are the dominant cause of both choice errors and of variations in response time (RT) from choice-to-choice. However, it soon became evident that within-choice noise is not by itself sufficient to enable models to provide a comprehensive account of choice behavior. In seminal work, Laming (1968) and Ratcliff (1978) demonstrated that accounting for not only the frequency with which different choices are made but also the distribution of RT for every type of choice requires the addition of effects due to choice-to-choice fluctuations (i.e., *between-choice noises*).

In a departure from the usual assumption, Brown and Heathcote (2005a) asked whether between-choice noises alone could provide a comprehensive account of simple-choice behavior. Although acknowledging that a range of extrinsic (e.g., stimulus) and intrinsic (e.g., neural) factors can cause within-choice noise, they proposed that the attendant behavioral effects might sometimes be small enough to neglect. In support of this simplifying approximation, which they described as “ballistic,” they demonstrated that a model with no within-choice noise could provide a detailed account of a broad range of benchmark simple-choice behaviors. Their model, the ballistic accumulator (BA), was further simplified by Brown and Heathcote (2008) into the linear ballistic accumulator (LBA). The LBA was shown to provide an account of benchmark phenomena on par with the BA while gaining considerably in ease of application because of greater mathematical and computational tractability.

Here we extend Brown and Heathcote’s (2005a, 2008) line of argument by developing an even more mathematically tractable evidence accumulation model that shares with the LBA the assumptions that accumulation is linear and deterministic. We first set the context for this development by reviewing the roles of different types of noise in evidence accumulation models and by defining a framework within which the LBA, and our new proposal, the Lognormal race (LNR), are special cases.

Next we motivate the LNR model’s Lognormal distribution assumption, derive mathematical results, and show that the LNR model, because of its simplicity, is required to explain speed-accuracy trade-offs in an unconventional way, via changes in evidence accumulation. Finally, we test and compare the LBA and LNR models by fitting them to behavioral data from a lexical-decision task (i.e., classifying a letter string as either a word or a non-word) reported by Wagenmaker et al.’s (2008, Experiment 1).

We focused on Wagenmaker et al.’s (2008) experiment because it produced a very large speed-accuracy trade-off using instructions that emphasized either response speed or response accuracy. Fitting methods developed by Donkin et al. (2011a) enabled us to systematically explore a large variety of LBA and LNR model parameterizations that instantiate different ways to quantitatively explain the speed-accuracy trade-off. We show that LNR model is able to provide an accurate description of the frequency of each choice and its associated RT distribution. We also show that the LBA model is only able to provide the same accurate description if it explains a large part of the observed speed-accuracy trade-off in the same unconventional way as the LNR.

## Sources of Noise in Evidence Accumulation Models

Early evidence accumulation models – random walks and their continuous analog, a diffusion process (e.g., Stone, 1960) – assumed only within-choice noise. However, such simple models are inadequate because they predict correct and error choices have identical RT distributions, whereas empirically correct and error RT differ in regular and often replicated ways. For example, when decision accuracy is stressed errors are slower than correct responses, but when decision speed is stressed this difference decreases and can even reverse (e.g., Ratcliff and Rouder, 1998). These limitations can be remedied by the addition of two sources of between-choice noise.

First, Laming (1968) showed that variability in the starting point of a random walk process causes fast errors. As the starting point determines the amount of evidence required for each choice, there is an attendant between-choice fluctuation in response bias. Second, Ratcliff (1978) accounted for the more commonly occurring slow errors in a diffusion model by allowing the mean rate of evidence accumulation to differ between trials. Between-choice noise in the mean rate of evidence accumulation also allows these models to escape a prediction that is clearly false for many choice tasks; that perfect accuracy can be achieved by a sufficient increase in the amount of evidence required to make a choice.

What are the causes of these types of between-choice noise? In Ratcliff’s (1978) application – episodic recognition memory – mean rate variation could plausibly be attributed to substantial differences in memorability between test items (words), as responses to different words were aggregated within experimental conditions. Subsequent research has shown that mean rate variation is also required to fit behavioral data from paradigms using homogenous test items within each experimental condition. This suggests there may be other causes of mean rate variations not related to item effects, such as choice-to-choice fluctuations in attention and arousal. Shadlen and Newsome (1998) provide a potential neural cause; they showed that correlations among the firing of neurons coding the same stimulus also cause choice-to-choice variations in mean spike-rates.

Sequential effects are the most commonly proposed cause of between-choice noise in the starting points of evidence accumulation (i.e., in the amount of evidence required for each choice). Simple-choice paradigms typically require participants to make a series of closely spaced decisions, so residual effects from previous decisions have been proposed as a source of start-point noise in cognitive (e.g., Brown et al., 2008) and neurophysiological (e.g., Gao et al., 2009) process models. Van Maanen et al. (2011) recently reported evidence that model-based estimates of choice-to-choice fluctuations in the amount of evidence required for a response are correlated with changes in hemodynamic responses in areas associated with response caution, the pre-supplementary motor area and anterior cingulate (see also Huettel et al., 2002).

The most widely and successfully applied evidence accumulation model, the Ratcliff diffusion model (RDM, see Ratcliff and McKoon, 2008, for a summary), owes its ability to provide a comprehensive account of decision behavior to the inclusion of between-choice noise in both start points and mean rates. More recently, a third type of between-choice noise, in the time to complete encoding and response production processes (denoted *Ter*). *Ter* noise was required by Ratcliff et al. (2004) to be able to enforce the assumption that word frequency selectively influences the rate of evidence accumulation in a lexical-decision task, as otherwise they could not account for systematic effects of word frequency on fast responses.

Race models constitute a second important class of evidence accumulation models (see Marley and Colonius, 1992, for an overview). In a race model each choice is represented by a separate accumulator, with the choice made corresponding to the first accumulator to hit its evidence boundary, and RT to the time required to do so. Like the diffusion model, early race models (e.g., Vickers, 1979) assumed a dominant role for within-choice noise and linear accumulation (i.e., equal weighting of samples taken earlier vs. later in the accumulation process). These simple assumptions were shown to be problematic because they predicted that the distribution of RT became more symmetric as overall RT slowed. Empirically, RT distributions show strong positive skew for all but the simplest and most rapid decisions (Luce, 1986).

This problem with race models was overcome by non-linear accumulation in Usher and McClelland’s (2001) Leaky Competitive Accumulator (LCA) model. Their model’s non-linear accumulation mechanisms were inspired by the fact that single-cell neural dynamics are commonly found to be “leaky” (i.e., firing rates return to baseline in the absence of input) and competitive (i.e., increased firing in one neuron can suppress firing in another). The interplay between these two types of non-linearity (i.e., either one or the other dominating) can result either in late-arriving evidence being more influential on the eventual choice (due to leakage) or in early arriving evidence being more influential (due to competition). Non-linear accumulation has also been proposed in a generalization of the class of diffusion models, an Ornstein–Uhlenbeck process, as part of Busemeyer and Townsend’s (1993) Decision Field Theory (DFT). Ratcliff and Smith (2004) claimed that this generalization was not required based on an analysis that estimated the degree of non-linearity from fits to data. However, more recent work by Leite and Ratcliff (2010), using the same type of analysis, found leakage to be necessary in non-competitive race models dominated by within-choice noise.

Brown and Heathcote’s (2005a, 2008) BA and LBA are race models that have their roots in models from both cognitive psychology and decision neuroscience. The BA is a race model identical in architecture and deterministic non-linear dynamics to Usher and McClelland’s (2001) LCA, but with only between-choice (start point and rate) noise. The LBA model removes two further components of the LCA, leakage in accumulation, and competition between accumulators. As a result accumulation is linear in the LBA and the level of evidence in one accumulator is independent of the level in other accumulators until one hits its boundary. At that time all other accumulators are inhibited, so that only one response is made. The overall architecture of the LBA is identical to Logan and Cowan’s (1984) horse-race model of the stop-signal paradigm. The LBA model’s assumption that inhibition plays a role after, rather than during, accumulation is largely consistent with Boucher et al.’s (2007) neurobiological findings in the stop-signal task.

The LBA’s assumption that accumulation is linear and deterministic, and that the rate characterizing this linear accumulation varies among choices according to a normal distribution, are shared with Carpenter’s (1981) LATER model. The LATER model has been widely applied to behavioral and neuroscience studies where responding is via eye-movements and the focus is on modeling (i.e., non-choice) RT. Ratcliff (2001) pointed out that LATER is unable to account for effects related to error responses in choice paradigms. The LBA differs from LATER in assuming that the distance from the starting point of accumulation to the boundary varies between trials according to a uniform distribution. This added assumption allows the LBA to account for error related phenomena, such as systematic differences between correct and error responses and speed-accuracy trade-offs.

As pointed out by Ratcliff (2001), within-choice noise models account for speed-accuracy trade-off because accumulation integrates out moment-to-moment fluctuations in evidence. In the LBA speed-accuracy trade-off can occur because accumulation integrates out response bias due to between-choice start-point noise. In both model classes between-choice rate noise also serves to limit the accuracy that can be achieved by increasing response caution. This provides one explanation of the observation that even very slow decisions can be inaccurate in some tasks.

It is important to clarify the meaning of the term “ballistic” as employed by Brown and Heathcote (2005a, 2008), as it is non-standard. It does not imply that, like a projectile fired from a cannon, the trajectory of evidence accumulation is entirely determined when initiated. Rather, it indicates a lack of within-choice noise. To illustrate this point, consider Brown and Heathcote’s (2005b) experiment using stimuli that briefly (e.g., for 90 ms) favored one choice, then switched to favor an alternative choice. They conceived of the rate of evidence accumulation as being able to change as a function of the stimulus change during a trial. Interference paradigms, such as the flanker task (e.g., Gratton et al., 1988), provide another case where it is likely that the input to the accumulation process is non-stationary (i.e., changes over time). Naturally, sensitivity to stimulus change is limited by the low-pass filtering imposed by sensory processes, and such sensitivity will also vary depending on attention-mediated selection of task relevant vs. irrelevant features. An example is provided by the global vs. local motion classification task with random-dot kinematogram stimuli used by Ho et al. (2009); they reported a successful application of a stationary-rate LBA model to global motion choices based on their rapidly time-varying stimuli.

## The Linear Deterministic Accumulation Framework

In this section we articulate a general framework for linear deterministic accumulation models. Within this framework particular models differ in the assumptions they make about the distributions followed by each type of between-choice noise. We begin by outlining the general framework, using the LBA model as an illustration, and then we develop a new model that makes different distributional assumptions, the LNR.

The LBA assumes a uniform distribution of start-point noise and normal distribution for rate noise. These assumptions were made both as a matter of convention (e.g., the same assumptions are made by the RDM) and mathematical convenience. Mathematically, they enable computationally tractable solutions for the density and cumulative density functions describing the distribution of times at which the evidence total first hits the boundary of a single accumulator. These two functions can then be easily combined to determine the likelihood of any given response at any given time from a set of one or more potential responses (i.e., for a race amongst any number of accumulators). A likelihood, which is not easily computed for alternative models such as the LCA and RDM, enables efficient model estimation, and so has facilitated applications of the LBA (see Donkin et al., 2011a, for a tutorial).

Equation 1 characterizes the time, *T*, for a single evidence total to accumulate to a boundary without the specific commitments to distributional assumptions made by Brown and Heathcote’s (2008) LBA.

(1) $$T=\frac{D}{V}$$

The numerator of the ratio in (1), *D* ≥ 0, indicates the distance between the starting point of evidence accumulation and the boundary, and (1) assumes that *T* is undefined if *V* ≤ 0. For the LBA, *D* ~ *B* + *U*(0, *A*), where “~” means “is distributed as,” *U*(0, *A*) indicates the uniform start-point distribution on the interval from 0 to *A*(*A* ≥ 0), and *B*(*B* ≥ 0) is the distance from the upper bound of the start-point distribution to the evidence boundary. The denominator, *V*, is the rate (velocity) of evidence accumulation. The LBA rate distribution is normal with a mean of *v* and standard deviation *sv*: *V* ~ *N*(*v*, *sv*).

### A lexical-decision task example

**Figure 1:**
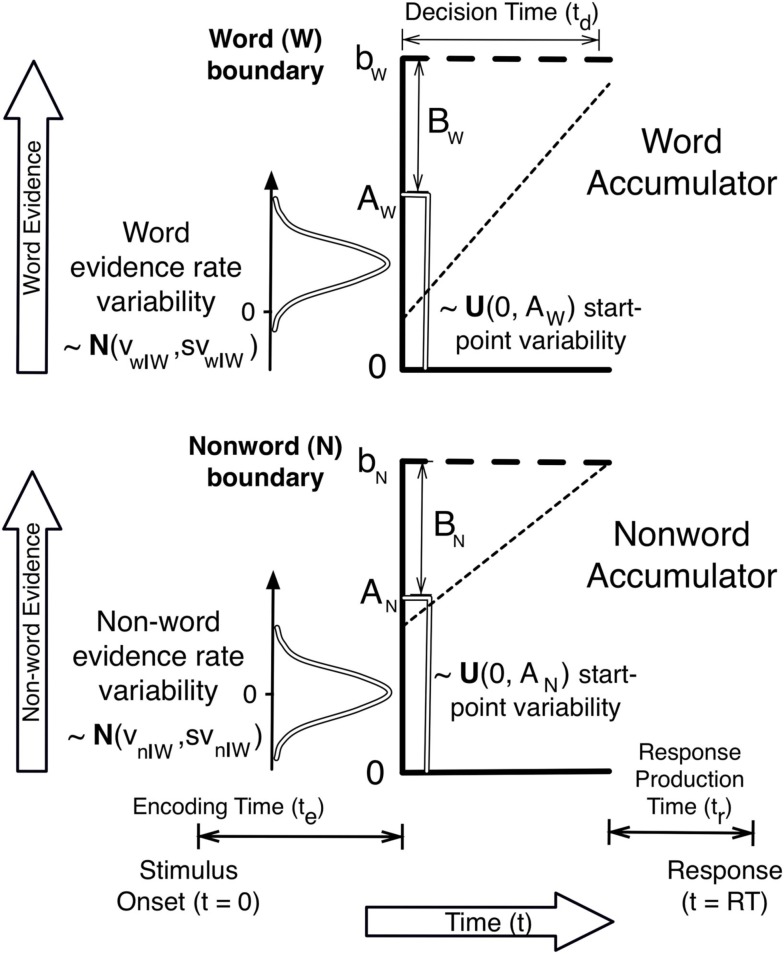
**A schematic illustration of an LBA model for the lexical-decision task with a word stimulus**.

Figure 1 illustrates an LBA model of a lexical-decision task, in which participants have to decide if a string of letters makes up a word. Figure 1 illustrates a trial in which the stimulus is a word, and so the rate distribution for the true (i.e., word) accumulator has a higher mean than the rate distribution for the false (i.e., non-word) accumulator. In this illustration the sampled rates (indicated by the slope of the dotted line) follow the same order as the mean rates, but a choice error (i.e., a non-word response) is made because the non-word accumulator hit its boundary first. The error occurs because the non-word accumulator starts with a higher level of evidence.

One way in which a speed-accuracy trade-off can be explained is illustrated by considering what would happen if the boundary were sufficiently increased in Figure 1; the higher rate of the word accumulator would eventually overcome the initial response bias in favor of the non-word accumulator and an accurate word response would be made. Past applications of the BA and LBA (Brown and Heathcote, 2005a, 2008) have assumed that this response caution based mechanism explains speed-accuracy trade-off caused by speed vs. accuracy emphasis instructions. However, it might also be explained by a change in the upper boundary of start-point noise distribution, *A*. If *A* decreased under accuracy emphasis responding would slow, as the average distance from start-point to boundary would increase, and become more accurate, as bias favoring the false accumulator would become weaker on average. The same is true of other accumulator models, such as LCA and DFT.

The overlap of the two rate distributions in Figure 1 illustrates why responding may not always be entirely accurate even with a very high boundary. On some trials a higher rate will be sampled for the incorrect (non-word) accumulator. In this case, if a quick correct response is not caused by response bias, an error will occur no matter how high the boundary is set. Although they have not conventionally done so, evidence accumulation models might also explain the effects of speed vs. accuracy emphasis through changes in mean rates and the level of rate noise (e.g., between-choice noise in the LBA and both within-choice and between-choice noise in the RDM, LCA, and DFT models). For example, if accuracy emphasis caused *v* and *sv* parameters to decrease equally for all accumulators, RT would slow (as it would take longer to hit an evidence boundary) and errors would decrease (due to a reduction in the overlap of the rate distributions).

Given that Wagenmakers et al. (2008) manipulated speed vs. accuracy emphasis between trial blocks participants may have had time to make global changes in factors like attention and arousal that might plausibly affect accumulation rates (Kleinsorge, 2001). Hence, rather than imposing one particular way of explaining the effects of emphasis, we took a more exploratory approach in fitting in Wagenmakers et al.’s data. That is we fit all of the different possible ways of explaining the effect of emphasis by allowing appropriate variation in the LBA’s *B*, *A*, *v*, and *sv* parameters.

### Multiple and contingent choice

The two-choice case illustrated in Figure 1 can be generalized to choice between any numbers of alternatives, where one accumulator corresponds to each alternative. Suppose the densities of *T* at time *t* for each of *i* = 1…*N* accumulators are denoted by *f*_i_(*t*) and the survivor functions (i.e., the complements of the cumulative densities) by *S*_i_(*t*). If the corresponding *D*_i_ and *V*_i_ are assumed independent between accumulators, as is the case for the LBA, the likelihood of response *i* at time *t*, where Π denotes a repeated product, is:

(2) $$T_{d}\left(i,t\right)=f_{i}\left(t\right)\prod_{j\neq i}S_{j}\left(t\right)$$

The expression for decision time (*T*_d_) in (2) describes what is sometimes called a defective density, a curve that integrates to a value less than or equal to one, where that value corresponds to the probability that response *i* wins the race.

Even more general, yet still computationally tractable, models can be derived based on race equations such as (2). For example, Eidels et al. (2010) describe a model in which binary responses are made contingent on logical relations between two stimuli as computed by four linear deterministic accumulators. This illustrates the considerable power and generality afforded by independent race models. As we discuss below, the LNR model extends the power of this approach by providing a tractable approach to including correlations between the inputs to accumulators.

### Residual time

It is necessary to make one further addition to the framework to describe observed behavior, an account of the “residual” component of RT not accounted for by decision time. This non-decision time is conventionally annotated “Ter,” an acronym for time (*T*) for encoding (*e*) and response (*r*). As illustrated in Figure 1, RT is typically assumed to be the sum of decision time and a residual time that does not vary as a function of either the stimulus or response. This invariance is reasonable in typical rapid choice paradigms where the difficulty of stimulus encoding and response production is fairly homogenous, but it need not always apply. For example, Karayanidis et al. (2009) reported large differences in residual time for fits of a diffusion model in a cued-task-switching paradigm as a function of whether the cue indicated a repeat or switch in task.

A second consideration related to residual time concerns whether it is a constant or whether it is variable. Although some variation in the processes causing residual time is highly likely, a constant will provide a reasonable approximation if that variation is small relative variation associated with decision time. For example, Smith (1995) reports evidence consistent with the standard deviation of motor production time in button-pressing tasks being of the order of 10 ms, which would mean it accounts for less than 1% of the overall variability in RT in even quite rapid choices. However, between-choice variability in residual time, in particular the sum of a constant and a uniform random deviate, has become standard in recent applications of the RDM (e.g., Ratcliff et al., 2004). We assume a constant residual time in the model tests described later, as that provides a substantial advantage in terms of computational speed for both the LBA and LNR. Although that suited the exploratory aims of these tests it does not indicate a commitment to residual time always being a constant in the general linear deterministic framework.

## The lognormal race model

We make two observations that provide some motivation for the LNR. First, as Brown and Heathcote (2008) note, it is possible for an LBA to fail to respond where no sampled rate is positive, although they found the probability of a non-response to be negligible in fits to data. However, non-responding is not a necessary characteristic of the general class of linear deterministic accumulators. Non-responding does not occur in the LNR model because the logarithm of the rate is assumed to have a normal distribution, and hence the rate for every accumulator has a (necessarily positive) Lognormal distribution. The same would be true of any other linear deterministic model that assumes a positive rate distribution.

Second, it turns out that the independence between accumulators assumed in (2) is not necessary to derive a computationally tractable expression for the LNR likelihood. Although widely made, we argue that the assumption of independence between accumulator inputs and/or between accumulator distances may be questionable in at least some circumstances, such as when evidence for each response alternative is derived from the same stimulus characteristics. In such circumstances, it is natural to assume choice-to-choice variations in stimuli will cause the inputs to different accumulators to be correlated to some degree, even if the input to each accumulator also contains some stimulus independent sources of noise. The Lognormal distributional assumption allows us to avoid the independence assumption without greatly increasing the computational cost of estimation.

The LNR model derives from the work of Ulrich and Miller (1993), who proposed that RT dynamics could be approximated by simple version of a “continuous flow” or “partial outputs” system (Schweikert, 1989; Townsend and Fikes, 1995). They examined a system that is time invariant (autonomous), in the sense that its rate of change does not depend directly on time, and that has no memory, in the sense that its rate of change is independent of its current state (activation). The latter property distinguishes this system from perhaps the most well known partial outputs model, McClelland’s (1979) cascade process. Ulrich and Miller derived the prediction of approximately Lognormal RT for a flow with stages characterized by a constant rate of change (i.e., linear accumulators). In the next section we briefly summarize their development and show how it can be used to model simple (i.e., non-choice) RT tasks (e.g., press a button when a light comes on). We then expand the development to account tasks where participants must choose between two or more responses.

### Simple response time

Suppose a flow is made up of *S* linear accumulator stages, with associated rates *v*_s_, *s* = 1‥*S*, the activation, *x*, of the terminal stage as a function of time, *t*, is given by *x*_S_ = *tV*, where *V* = Π_s=1‥Sv__s_. The flow can be approximated as being “lumped,” in the sense that transmission occurs instantaneously from the initial to a terminal stage with any delay confined to a time, *t*_1_, between stimulus presentation and the time at which that presentation begins to effect activation in the flow. Without loss of generality it can also be assumed that the sensory input to the first stage is *x*_0_ = 1, as any differences in input magnitude can be absorbed in to the rate of the first stage. The terminal stage is a unit that represents a response, in the sense that a response is initiated when the activation of a terminal unit travels a distance *D* from its initial state.

The time taken to traverse that distance from the time that sensory processing commences has the same form, *T* = *D*/*V*, as (1) from the general framework. Ulrich and Miller’s (1993) system is sufficient to model simple RT given the specification of the time required for response production, *t*_2_. They assumed stage rates vary from choice-to-choice with distributions, *Z*_s_, that are positive, independent and identically distributed, with finite first and second moments, μ_s_ and $\sigma_{\text{s}}^{\text{2}}.$ It follows from the central limit theorem that the rate distribution *V* ~ exp(Σ_s=1‥S_ *Z*_s_) can be approximated by LN(μ_V_, $\sigma_{\text{V}}^{2}$), where LN indicates a Lognormal distribution with a mean, μ_V_, and variance, $\sigma_{\text{V}}^{2}$ that equal the sums of the first and second central moments, respectively, of the *Z*_s_.

We define the LNR as *a model made up of one or more racing Lognormal accumulators* (i.e., accumulators for which *T* ~ LN). Of course, when there is only one accumulator there is not really a race, but the single accumulator model is useful for modeling simple RT. Whether motivated by Ulrich and Miller’s (1993) flow argument, or simply made as an *ad hoc* assumption, a Lognormal distribution for the rate of evidence accumulation is very mathematically convenient in the linear deterministic accumulation framework. This is so because both the inverse of a Lognormal variable, and the product of independent Lognormal variables, also have a Lognormal distribution. If one assumes, as did Ulrich and Miller, that the distance *D* is a constant, it follows that *T* ~ LN(μ, σ^2^), where μ = ln(*D*) − μ_V_ and $\sigma^{2}=\sigma_{\text{V}}^{\text{2}}.$Consequently, simple RT is predicted to have a shifted Lognormal distribution, where the shift, which defines the lower bound of the distribution, equals *t*_1_ + *t*_2_. Note that if accumulators in which *T* ~ LN are embedded in a race architecture, choice RT is also predicted to be Lognormal in the limit of high accuracy. This occurs because when accuracy is high the race has no effect on RT distribution because one accumulator (representing the correct response) always wins.

Consistent with these predictions, the shifted Lognormal has a long history of use to describe simple and choice RT distributions (e.g., Woodworth and Schlosberg, 1954), which it does with an accuracy that has been found to be on par with the most widely used descriptive model, the ExGaussian distribution (Ratcliff and Murdock, 1976). The Lognormal distribution is bounded below by zero (*x* > 0) with density:

(3) $$f\left(x,\mu ,\sigma \right)=\frac{1}{x\sigma \sqrt{2\pi }}{\text{e}}^{-\frac{1}{2}{\left(\frac{1n\left(x\right)-\mu }{\sigma }\right)}^{2}}$$

The Lognormal survivor function (*S*) can be expressed in terms of the standard normal cumulative distribution, Φ, which has rapidly computable approximations:

(4) $$S\left(x,\mu ,\sigma \right)=1-\Phi \left(\frac{1n\left(x\right)-\mu }{\sigma }\right)$$

**Figure 2:**
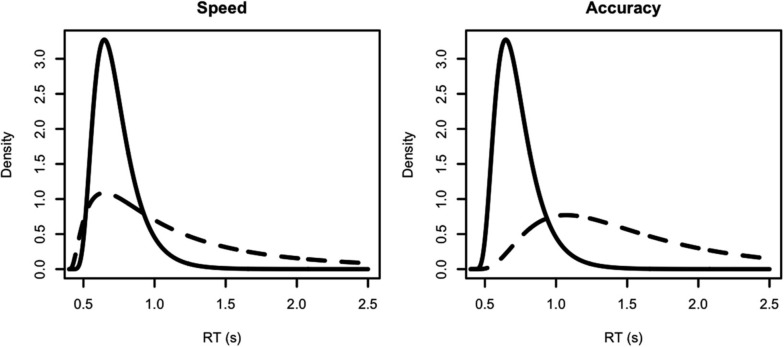
**Examples of independent Lognormal distributions, all with shift þeta = 0.4 s**. Solid lines represent the true (correct response) accumulator distribution, dashed lines the false (error response) accumulator distribution. The left panel represents a speed-emphasis condition, the right panel an accuracy emphasis condition. For the speed condition the (μ, σ^2^) parameters for each density are: solid line (−1.2, 0.2), dashed line (−0.5, 0.9). The corresponding mean RTs for correct and error responses are 0.7 and 0.64 s (fast errors) with 75% accuracy. For the accuracy condition the (μ, σ^2^) parameters for each density are: solid line (−1, 0.2), dashed line (0, 0.4). The corresponding mean RTs for correct and error responses are 0.78 and 0.84 s (slow errors) with 90% accuracy.

To allow for a lower bound greater than zero a shift parameter, 0 < θ < min(*x*), can be added by substituting (*x* − θ) for *x* in (3) and (4). Figure 2 shows four examples of the shifted Lognormal density.

It is important to observe that this Lognormal form is not uniquely predicted by assuming that distance is a constant. It also follows if distance is a random variable that also has a Lognormal distribution. That is, if *D* ~ exp(*Z*_D_), where *Z*_D_ ~ *N*(μ_D_, $\sigma_{\text{D}}^{2}$), then *T* ~ LN(μ, σ^2^), where μ = μ_D_ − μ_V_ and $\sigma^{2}=\sigma_{\text{D}}^{2}+\sigma_{\text{V}}^{2}.$ Similarly, if *V* is a constant and *D* ~ exp(*Z*_D_) *T* ~ LN(μ, σ^2^), where μ = μ_D_ − ln(*V*) and $\sigma^{2}=\sigma_{\text{D}}^{2}.$ These observations indicate that it is not possible to determine which of *D* of *V* are constant or Lognormal, or whether both are Lognormal, based only on the form of the distribution of *T*.

In contrast, a Lognormal distribution for *T* does not follow when *D* has alternative distributional forms. This is true even when the two cases discussed so far are combined, so that *D* has a shifted Lognormal form, *D* ~ *d* + exp(*N*(μ_D_,$\sigma_{\text{D}}^{2}$)), where *d* is a constant. Allowing distance to have a shifted Lognormal distribution would provide similar flexibility to the LBA, where the analogous condition is that there is some distance greater than zero between the top of the start-point distribution and the boundary (i.e., *B* > 0). This type of flexibility is necessary for the LBA to be able to account for responding under accuracy emphasis. That is, good fits of the LBA can be obtained with small values of *B* under speed emphasis (Brown and Heathcote, 2008), but in most other situations estimates of *B* are substantially greater than zero. The fits of the LNR model reported below enable us to test whether it fails because it lacks similar flexibility (i.e., it does not allow distance to be both variable and to have a minimum value greater than zero).

### Choice response time

As in the LBA, the LNR model of *N*-choice paradigms assumes a race among *n* = 1…*N* linear accumulators. The winning unit triggers its corresponding response production process and effectively inhibits all other choice units instantaneously, so only one response is made. In this section we explicitly derive the likelihood for a two-choice LNR model and point out the relatively straightforward extension required for the *N*-choice case. We do so with a quite general characterization of the sets of rate and distance parameters defined over choice units in terms of arbitrary finite variance-covariance matrices. We do, however, assume that distance and rate variation can be approximated as independent, which is plausible given they originate from different sources. We also explicitly develop results for an LNR model in which both distances and rates have Lognormal distributions, $D\sim {\text{e}}^{N\left(\mu_{d},\sigma_{d}^{2}\right)}$ and$V\sim {\text{e}}^{N\left(\mu_{v},\sigma_{v}^{2}\right)},$ an so:

(5) $$T=\frac{D}{V}\sim {\text{e}}^{N\left(\mu_{d}-\mu_{v},\sigma_{d}^{2}+\sigma_{v}^{2}\right)}={\text{e}}^{N\left(\mu ,\sigma^{2}\right)}$$

Note that in (5) and in the following, results for the case where distance is a constant are obtained by assuming parameters related to variability in distance (e.g., σ_d_) are set to zero.

Consider a race between two accumulators, so we now have two-vectors of random variable for distances and rates. Distances might be correlated between the accumulators and rates might be correlated between the accumulators, but distances and rates are assumed not to be correlated within an accumulator, or between accumulators. Given MVN (**μ**, **Σ**) denotes a multivariate normal random variable with mean vector **μ** and variance-covariance matrix **Σ**:

(6) $$\text{d}∼e^{\text{MVN}({\text{μ}}_{d}-\sum_{d})},\;\sum_{d}\left[\begin{array}{cc} \sigma_{d1}^{2} & \sigma_{d1.d2}^{2}\\ \sigma_{d1.d2}^{2} & \sigma_{d2}^{2} \end{array}\right]$$

(7) $$\text{v}∼{\text{e}}^{\text{MVN}({\text{μ}}_{v}-\sum_{v})},\;\sum_{v}\left[\begin{array}{cc} \sigma_{v1}^{2} & \sigma_{v1.v2}^{2}\\ \sigma_{v1.v2}^{2} & \sigma_{v2}^{2} \end{array}\right]$$

Using the fact that a difference between two independent multivariate normal random variables is also multivariate normal, where the mean vector of the difference is the difference of the mean vectors, and the variance/covariance matrix of the difference is the sum of the variance/covariance matrices, we can write:

(8) $$\begin{array}{c} T∼e^{\text{MVN}(\text{μ},\Sigma )\;},\text{μ}={\text{μ}}_{d}-{\text{μ}}_{v},\\ \sum =\left[\begin{array}{cc} \sigma_{d1}^{2}+\sigma_{v1}^{2} & \sigma_{d1.d2}^{2}+\sigma_{v1.v2}^{2}\\ \sigma_{d1.d2}^{2}+\sigma_{v1.v2}^{2} & \sigma_{d2}^{2}+\sigma_{v2}^{2} \end{array}\right]=\left[\begin{array}{cc} \sigma_{1}^{2} & \sigma_{1.2}^{2}\\ \sigma_{1.2}^{2} & \sigma_{2}^{2} \end{array}\right] \end{array}$$

In order to get the likelihood that, say, accumulator 2 wins the race at time *x* (i.e., hits its boundary at *T* = *x* and accumulator 1 has not yet hit its boundary) we need to multiply the marginal density of accumulator 2 by the conditional survivor function of accumulator 1. The marginal distribution of accumulator 2 is Lognormal, exp[*N*(μ_2_,$\sigma_{2}^{2}$)], as is the conditional distribution of accumulator 1. That is, the distribution of *T*_1_ conditional on *T*_2_ = *x* is Lognormal. Denoting the correlation $\rho =\sigma_{12}^{2}∕\left(\sigma_{1}\sigma_{2}\right):$

(9) $$T_{1}|\left(T_{2}=x\right)\sim e^{N}\left(\mu_{1}+\left(\sigma_{1}∕\sigma_{2}\right)\rho \left(\ln\left(x\right)-\mu_{2}\right),\;\left(1-\rho^{2}\right)\sigma_{1}^{2}\right)$$

For the case of *N* > 2 accumulators the required conditional is multivariate Lognormal. In the *N* = 2 accumulator case:

(10) $$L_{2}\left(x\right)=f\left(x,\mu_{2},\sigma_{2}\right)S\left(x,\mu_{1}+\frac{\sigma_{1}}{\sigma_{2}}\rho \left(ln\left(x\right)-\mu_{2}\right),\left(1-\rho^{2}\right)\sigma_{1}^{2}\right)$$

The likelihood that accumulator 1 finishes first at time *T* = *x* is obtained by exchanging indices in (10).

### Correct and error-response speed

Consistent with many other studies (e.g., Ratcliff and Rouder, 1998), Wagenmaker et al.’s (2008) speed vs. accuracy emphasis manipulation systematically affected the relative speed of correct and error responses; error responses were slower than correct responses under accuracy emphasis and equal or faster under speed emphasis. In order to understand the LNR and LBA fitting results that we report in the next section, it is useful to illustrate how each model can produce fast and slow errors. This illustration particularly focuses on the variability parameters, which play a more important role in the LNR than have analogous variability parameters in previous applications of the LBA.

The LBA predicts fast errors when the distance *B* (see Figure 1) is smaller. Errors are fast in this case because incorrect responses mostly occur when there is a strong initial bias toward the wrong response. This bias can occur on some trials due to random variation in the starting points of the accumulators. Hence, even if the error accumulator has a slow rate it can quickly achieve its boundary, and so produce a fast response. No similar characterization of fast errors is possible for the LNR model as bias (distance) and rate effects combine additively to determine distribution shape.

However, the LNR model can still produce fast errors, as illustrated by the left panel of Figure 2, which simulates results from a speed-emphasis condition. In this panel the variance parameter for the false (error response) accumulator is twice that of the true (correct response) accumulator. Despite having a much slower mean, and consequently only winning 25% of races (i.e., a 25% error rate), its greater variance causes the false accumulator to produce fast responses when it wins the race. This can be seen as the higher density for the false accumulator (dashed line) than the true accumulator (solid line) on the left of the RT distributions. As a result, when errors occur they tend to be faster than correct responses (0.64 vs. 0.7 s in the example). The right-hand panel of Figure 2 shows that greater false than true accumulator variance does not always produce fast errors. In this example performance is slower overall and more accurate (10% errors) with the mean for error responses being greater than for correct responses (0.84 vs. 0.78 s in the example).

These considerations suggest that the LNR will require different variances for true and false accumulators in order to fit manipulations that affect the relative speed of correct and error responses. Clearly these differences must arise from differences in rate variance, as whether an accumulator represents a correct or error response is determined by the stimulus. We consider how such differences might arise after reporting the results of model fitting in the next section.

Note that fast errors could also occur in the LBA if the *sv* (rate standard deviation) parameter is greater for false than true accumulators. This possibility has not been tested before because previous applications of the LBA have either assumed a fixed value of *sv* (e.g., unity), or that the same estimated value applies for all accumulators and experimental conditions. These assumptions about *sv* were motivated by the fact that an accumulation-related LBA parameter must be fixed in order for the model to be identifiable. However, in a design such as was used by Wagenmakers et al. (2008) identifiability requires only that one parameter value be fixed for one accumulator in one condition (see Donkin et al., 2009b). The next section reports fits of LBA models that only place this minimal constraint on *sv* so that we can examine all potential explanations for fast errors.

## Model Testing

We fit the LBA and LNR models to data from Wagenmaker et al.’s (2008) experiment one, where participants made decisions about whether a string of letters constituted a word. These lexical decisions were made about four types of stimuli, non-words (nw) and high-frequency (hf), low-frequency (lf), and very low-frequency (vlf) words. Participants made decisions either under speed or accuracy emphasis instructions in different experimental blocks. Accuracy blocks were preceded by the message “Try to respond accurately” and “ERROR” was displayed after each wrong response. Speed blocks were preceded by the message “Try to respond accurately” and “TOO SLOW” was displayed after each response slower than 0.75 s. We report analyses of data from 17 participants (31,412 data points) in their Experiment 1, including the 15 participants analyzed in Wagenmakers et al. (2008) and two extras (we thank Eric-Jan Wagenmakers for supplying this data).

### Fitting methods

As we examined a large number of parameterizations of each model that varied widely in the number of estimated parameters, we compared fits both within and between model types using the AIC and BIC model selection criteria (Myung and Pitt, 1997). These criteria measure badness-of-fit using twice minus the log-likelihood, which we will call the deviance (*D*). A model is selected from amongst a set of models if it has the lowest AIC or BIC. Both criteria add to the deviance a penalty that increases with the number of parameters estimated to obtain a fit. Hence, better fitting models (i.e., with a smaller *D*) that have a larger numbers of parameters may not be selected if the extra parameters do not produce a sufficiently large improvement in fit (i.e., reduction in *D*). We consider both AIC and BIC criteria as they have different merits flowing from the underlying quantities that they approximate (see Burnham and Anderson, 2004; Vrieze, 2012), with BIC generally preferring simpler models.

Our LNR models assumed there were no correlation among rates or among distances, so fits were obtained using the correspondingly simplified from of (10) corresponding to (2), i.e., $F_{1}\left(x\right)=f\left(x,\mu_{1},\sigma_{1}\right)S\left(x,\mu_{2},\sigma_{2}\right)$ and $F_{2}\left(x\right)=f\left(x,\mu_{2},\sigma_{2}\right)S\left(x,\mu_{1},\sigma_{1}\right).$ Although we argued correlations are plausible, in this initial exploration we fixed them at zero for three reasons: (1) to make understanding the LNR model easier, (2) to determine if correlation is necessary to accommodate the benchmark effects present in Wagenmaker et al.’s (2008) data, and (3) to make the LNR and LBA models more comparable.

As well as the standard LBA model described by Brown and Heathcote (2008) we also fit two variations that guaranteed a response on every trial. In the first variation rates for all accumulators were guaranteed to be positive on every trial by sampling them from univariate normal distributions truncated below at zero, and in the second at least one sampled rate was guaranteed to be positive on every trial by sampling the rates from a truncated multivariate normal distribution^1^. All of these LBA models produced similar fits and parameter estimates, so we report fits from the second variant for two reasons: (1) it is most directly comparable to the LNR model, which also has positive rates for all accumulators on every trial and (2) it is of interest in itself as an alternative to the LNR for solving the issue of potential non-responding in the original LBA model.

As it is not our focus here we do not provide a direct comparison with fits of the RDM reported by Wagenmaker et al.’s (2008). Note, however, that Donkin et al. (2011b) found similar quality fits to this data for Ratcliff diffusion and LBA models with similar numbers of parameters. In this comparison both parameterizations were strongly constrained based on past findings about these models. In contrast, because past findings are not available for the LNR model, and our aim here is exploratory, our most complex LNR model was very flexibly parameterized. It freely estimated both μ (mean) and σ^2^ (variance) parameters over a false vs. true accumulator factor (*C*), a stimulus type factor (*W*) with four levels (hf, lf, vlf, and nw), and an instruction emphasis factor (*E*) with two levels (speed vs. accuracy), so in total there are potentially 2 × 4 × 2 = 16 estimates of each type. Note that *C* factor represents whether, for a given stimulus, a parameter corresponds to the accumulator for the correct (true) response (i.e., the word accumulator for a word stimulus or the non-word accumulator for a non-word stimulus) or for an error (false) response (i.e., the word accumulator for a non-word stimulus or the non-word accumulator for a word stimulus).

In order to allow similar flexibility for the LBA, and so that we can compare effects on analogous parameters, we report fits of an LBA model with a less constrained parameterization than is conventional. The LBA *B* and *A* parameters were allowed to vary with a word vs. non-word response accumulator factor (lR, standing for “latent response”), in order to accommodate response bias. We also allowed these parameters to vary as a function of the speed vs. accuracy emphasis factor (*E*), as the analogous quantity in the LNR model, the mean and variance of distance, have the same freedom to vary as components of the Lognormal mean and variance parameters. LBA rate mean (*v*) and variability (*sv*) were allowed to vary with *C*, *E*, and *W* as the analogous LNR quantities, the rate mean and variance, are components of the LNR mean and variance parameters.

We also allowed residual time to vary with emphasis instructions (*E*) for both models as this was favored by AIC and BIC model selection in most cases (see Rinkenauer et al., 2004, for evidence supporting an effect of emphasis instructions on response production). We denote residual time as *t*0, as it corresponds to the lower bound of the distribution of RT. We used this notation rather than by the conventional *Ter* notation because, at least in the flow interpretation of the LNR, stimulus-encoding time might be seen as part of the accumulation process, in which case *t*0 should be thought of as the sum of response production time and the “dead-time” between stimulus onset and the first stimulus contingent change in the firing rates of sensory neurons.

In summary, there were 34 estimated LNR parameters (16 each for μ and σ^2^ and two residual time parameters) compared to 41 LBA parameters (16 *v*, 15 *sv*, 4 *B* and 4*A*, and two residual time parameters). Note that we fixed the intercept of *sv* estimates at one (for the false accumulator in the accuracy condition for high-frequency words) to make the LBA model identifiable, so there are 15 rather than 16 estimated *sv* parameters. In order to compactly refer to models we useR (R Development Core Team, 2012) linear model notation adapted to our multiple parameter-type setting. For example the notation *B* ~ lR*E indicates estimation of the main effects of lR and *E*, and their interaction (lR × *E*). Similarly *v* ~ E*W*C indicates estimation of the three main effects, three two-way interactions (*E* *×* *C*, *E* *×* *W*, and *W* *×* *C*), and one three-way interaction (*E* *×* *W* *×* *C*). Using “&” to indicate a join between parameterizations for different parameter types, we denote the 34 parameter LNR model as μ ~ E*W*C & σ^2^~E*W*C & *t*0 ~ *E* and the 41 parameter LBA model as *B* ~ lR*E & *A* ~ lR*E & *v* ~ E*W*C & *sv* ~ E*W*C & *t*0 ~ *E*.

The models were fit to each participant’s data separately using the exact maximum-likelihood-based methods described in detail in Donkin et al. (2011a)^2^. This method fits a hierarchy of simplified models that are special cases of the most complex (“top”) model (i.e., the 34 parameter LNR model and the 41 parameter LBA model). The simplest LNR model in the hierarchy estimated the same parameters of each type for all conditions, except that it allowed different mean rate parameters for true and false accumulators (i.e., model μ ~ *C* & σ^2^ ~ 1 & *t*0 ~ 1, where “~1” indicates an intercept-only estimate). Similarly, the simplest LBA model allowed only mean rate to differ between true and false accumulators (*B* ~ 1 & *A* ~ 1 & *v* ~ *C* & *sv* ~ 1 & *t*0 ~ 1).

Best fitting parameters for the simplest models were used as starting points when searching for best fits of models that estimated the effect of one extra factor for one type of parameter. Best fits for these models were used in turn as starting points for fits of models that estimated one additional factor, and so on up to the most complex model. For example the simplest LNR model, μ ~ *C* & σ^2^ ~ 1 & *t*0 ~ 1, provides a start point for six models: μ ~ W*C & σ^2^ ~ 1 & *t*0 ~ 1, μ ~ E*C & σ^2^ ~ 1 & *t*0 ~ 1, μ ~ *C* & σ^2^ ~ *C* & *t*0 ~ 1, μ ~ *C* & σ^2^ ~ *W* & *t*0 ~ 1, μ ~ *C* & σ^2^ ~ *E* & *t*0 ~ 1, and μ ~ *C* & σ^2^ ~ 1 & *t*0 ~ *E*. One or more of these (nested) models then provided start points for further (nesting) models with three free factors and so on up to the top model.

The aim of fitting nesting models from immediately nested models is to avoid getting stuck in local minima and so to obtain true maximum-likelihood fits for more complicated models by fitting them from a variety of plausible starting points. We cannot prove that the method always achieves its aim but have found it does so in simulation studies fitting model of similar complexity to those examined here when using similar sample sizes to the data examined here. In contrast, we have found other less thorough methods often fail badly, and although hand tuning can often remedy such problems that is not feasible when exploring large sets of models. For example, here we fit all possible combinations of factors for different parameters, resulting in 64 LNR model and 1024 LBA models per participant (i.e., 18,496 models in total over 17 participants) and this was done for a number of variants (e.g., different types of LBA models and different levels of response contamination).

In total the results reported here were based on 193 LNR fits per participant and 5121 LBA fits per participant (90,338 fits in total). The R (R Development Core Team, 2012) *optim* function was used for each fit with default settings. It was repeatedly applied (starting each time from the previous solution) until the log-likelihood increased by less than 0.1. All model hierarchies were checked to ensure that nested models had an equal or higher deviance (within numerical tolerance) than nesting models, and any exceptions corrected by refitting.

### Model selection

Table 1 reports badness-of-fit (deviance) and AIC and BIC model selection results relative to the best model according to that statistic (i.e., the model with a zero in the corresponding column of Table 1). The table reports model selection results for three hierarchies. The first two are the full hierarchies below the top LBA (*B* ~ lR*E & *A* ~ lR*E & *v* ~ E*W*C & *sv* ~ E*W*C & *t*0 ~ *E*) and LNR (μ ~ E*W*C & σ^2^ ~ E*W*C & *t*0 ~ *E*) models. The third hierarchy consists of all models nested within an LBA model that enforces the conventional assumption that speed vs. accuracy emphasis does not influence evidence-rate-related parameters (i.e., *v* and *sv*): *B* ~ lR*E & *A* ~ lR*E & *v* ~ W*C & *sv* ~ W*C & *t*0 ~ *E*. We call this set of models the “conventional LBA” hierarchy, although it is still somewhat more flexible than most previous LBA applications as it allows changes in the *A* as well as the *B* parameter with emphasis.

**Table 1:** **Model selection statistics for the LBA and LNR top models and the conventional LBA top model (upper section) and the best AIC (middle section) and BIC (bottom section) model in each hierarchy**.

Model	NP	Δ*D*	ΔAIC	ΔBIC
*B* ~ lR*E & *A* ~ lR*E & *v* ~ E*W*C & *sv* ~ E*W*C & *t*0 ~ *E*	41	0	15	210
μ ~ E*W*C & σ^2^ ~ E*W*C & *t*0 ~ *E*	34	19	20	156
*B* ~ lR*E & *A* ~ lR*E & *v* ~ W*C & *sv* ~ W*C & *t*0 ~ *E*	25	68	51	112
*B* ~ lR*E & *A* ~ *E* & *v* ~ W*C & *sv* ~ E*C & *t*0 ~ *E*	19	29	0	11
μ ~ E*W*C & σ^2^ ~ E*C & *t*0 ~ *E*	22	36	13	49
*B* ~ lR*E & *A* ~ lR*E & *v* ~ W*C & *sv* ~ *C* & *t*0 ~ *E*	19	79	50	61
*B* ~ *E* & *A* ~ 1 & *v* ~ W*C & *sv* ~ E*C & *t*0 ~ 1	15	59	22	0
μ ~ E*W*C & σ^2^ ~ E*C & *t*0 ~ *E*	22	36	13	49
*B* ~ lR*E & *A* ~ *E* & *v* ~ W*C & *sv* ~ *C* & *t*0 ~ *E*	17	83	51	45

*NP, number of parameters per participant; Δ*D*, *D* − min(*D*), where *D* indicates deviance; ΔBIC, BIC − min(BIC); ΔAIC, AIC − min(AIC). *D*, BIC, and AIC values are summed over participants then divided by number of participants*.

Table 1 reports results for the most flexible model in each hierarchy its upper section, results for the best (smallest) AIC model in each hierarchy in the middle section, and results for the best (smallest) BIC model in each hierarchy in its lower section. Within each section the first two models are from the full LBA and LNR hierarchies, respectively, and the last model is from the conventional LBA hierarchy. As might be expected, the full LBA model, with the largest number of parameters, has the best fit (lowest deviance). Models from this same LBA hierarchy were also selected by AIC and BIC. Note that this is not because the selected models are more flexible as indexed by number of parameters; the best overall models have the same number parameters, or fewer, than the best models from the other hierarchies. Selection between the conventional LBA and LNR hierarchies is more equivocal, the LNR clearly wins on AIC but just looses on BIC.

The AIC and BIC results from the full LBA hierarchy are consistent in selecting the *v* ~ W*C and *sv* ~ E*C parameterization for rate-related parameters. That is, there is a selective influence of stimulus type on mean rate and selective influence of emphasis on rate variability, with true and false accumulators differing on both types of parameter. The conventional explanation of emphasis effects in terms of the boundary (*B*) is also consistently supported. However, an effect of emphasis on start-point noise (*A*) receives support only from AIC, indicating it has a weaker effect. The same is true of response bias (lR) effects on *B*, and there is no support for any effect of response bias on *A*. Support for an effect of emphasis on residual time (*t*0) was also inconsistent.

Model selection for the conventional LBA hierarchy produces results largely consistent with those for the full hierarchy. Stimulus type has a selective influence on mean rate (*v*), with true and false accumulators differing on both types of rate parameter. Both criteria support an effect of emphasis on the *B*, *A*, and *t*0 parameters, consistent with these parameters making up some of the fit provided by the *sv* parameter in the full hierarchy. Stronger response-bias effects, particularly on the *B* parameter, are also evident.

Within the LNR hierarchy both AIC and BIC pick the same model, which drops only the effect of stimulus type on variance relative to the full model. These results are largely consistent with effects selections for analogous parameters in the full LBA hierarchy (i.e., neglecting distance effects μ is analogous to −ln(*v*) and σ to *sv*), in that stimulus type selectively influences μ and both μ and σ differ between true and false accumulators. Two differences are that emphasis affects both μ and σ, not just the variability parameter as in the LBA, and it also consistently affects *t*0.

In summary, for the LBA, model selection results indicate that, in contrast to the conventional assumption, the speed vs. accuracy emphasis effect is best explained when rate variability changes with emphasis. Model selection also supports a role for the conventional boundary-based mechanism, but less so for the start-point based mechanism. Further, in contrast to previous applications where only the mean rate has been allowed to differ between correct and false accumulators, there was clear support for a difference in rate variability between accumulators. These results show that the LBA, at least to some degree, uses the same mechanism as the LNR to explain differences in the relative speed of correct and error responses, differences in the variability associated with true and false accumulators. The classes of models differ, at least in terms of the AIC and BIC selected models, in that the LNR mean parameter also has a role in explaining the emphasis effect.

### Model fit

**Figure 3:**
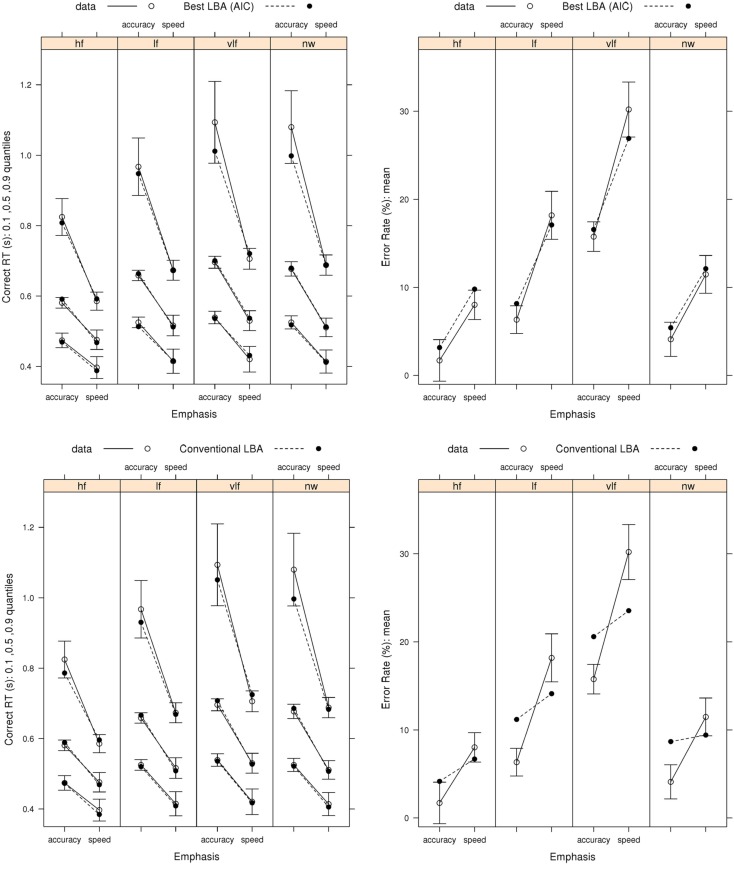

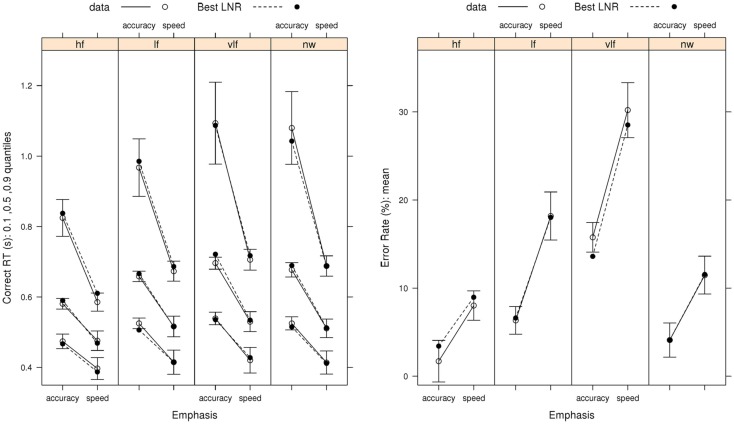
Observed RT distributions (10th, 50th, and 90th percentiles, left column) and error rates (right column) for Wagenmaker et al.’s (2008) Experiment 1 with bias-corrected within-subject 95% confidence intervals (Morey, 2008), and fits averaged over participants for the best (selected by AIC and BIC) LNR (μ ~ E*W*C & σ^2^ ~ E*C & *t*0 ~ *E*) and best (selected by AIC) LBA (*B ~ *lR*E & *A ~ E* & v ~ W*C & sv ~ E*C & *t*0* ~ E*) models and the top conventional LBA (*B ~ *lR*E & *A ~ *lR*E & v ~ W*C & sv ~ W*C & *t*0* ~ E*) model: hf, high-frequency words; lf, low-frequency words, vlf, very low-frequency words; nw, non-words.

**Figure 4:**
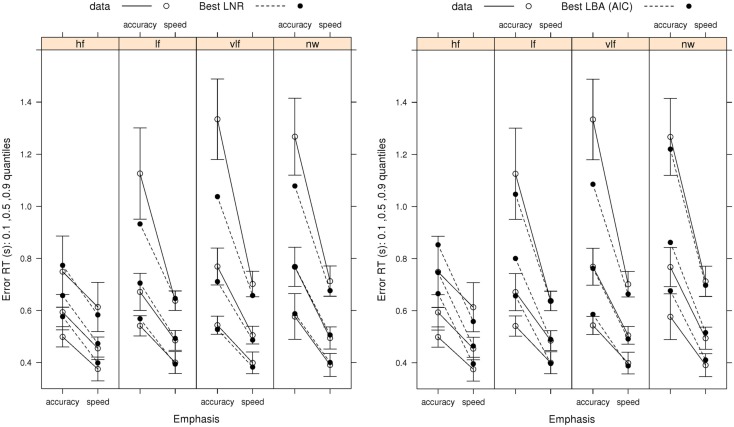
Observed error-response RT distributions (10th, 50th, and 90th percentiles) Wagenmaker et al.’s (2008) Experiment 1 with bias-corrected within-subject 95% confidence intervals (Morey, 2008), and fits averaged over participants for the best (selected by AIC and BIC) LNR (μ ~ E*W*C & σ^2^~E*C & *t*0 ~ *E*) and best (selected by AIC) LBA (*B ~ *lR*E & *A ~ E* & v ~ W*C & sv ~ E*C & *t*0* ~ E*) models: hf, high-frequency words; lf, low-frequency words; vlf, very low-frequency words; nw, non-words.

We focus on the fit of the model selected by both AIC and BIC from the full LNR hierarchy and by AIC for the LBA hierarchy in order to determine how well these relatively simple models capture the pattern of effects on correct RT and error rates (Figure 3) and error RT (Figure 4). The ability of the models to capture RT distribution is indicated by displaying results for the 10th, 50th, and 90th percentiles^3^ of RT distribution. Fits to intermediate percentiles are of similar quality to the percentiles shown, and are omitted for display clarity.

The goodness-of-fit figures average corresponding observed and predicted values over participants. Predicted values corresponding to each observed RT were obtained by simulating 100 times as many values as were observed. For example, if there were 100 correct and 9 error responses, 10,000 correct and 900 error RTs were simulated and predictions obtained from the order statistics of the simulated data (e.g., the fastest of the 90 error RTs was assumed to correspond to the 10th percentile of the simulated sample; in general the *i*th ordered observation from *N* was equated with the *i*/(*N* + 1) quantile of the simulated data). Proportion correct was calculated from the full set of samples required to obtain the predicted RT values. Observed and predicted values were treated in the same way to get the averages in the figures; RT averages were calculated omitting missing values.

Figure 3 shows that the best LNR and AIC-best LBA models accurately capture correct RT distribution. The simpler BIC-best LBA model, whose results are not shown, provided an equally accurate account of correct RT to the AIC-best LBA model. The word frequency manipulation provides a strong test of the simplifying assumption, which we made in fitting both the LNR and LBA models, that residual time is a constant, given the Ratcliff diffusion requires variable residual time to account for the effect of word frequency on fast correct responses (i.e., the 10th percentile). Clearly neither model requires variation in residual time to provide a very accurate account of fast correct responses.

Figure 3 shows that the best LNR model accurately captures error rates, although it slightly under-predicts the accuracy-speed effect for high-frequency words, although overall it captures 94% of the average difference. The AIC-best LBA model does not perform quite as well, capturing 83% of the average difference. Although this might be attributed to the AIC-best LBA model being simpler (19 parameters) than the best LNR model (22 parameters), the more complicated top LBA model (41 parameters) does not do much better (86%). The simpler BIC-best LBA model (15 parameters), which drops the effect of emphasis on *A*, does considerably worse (67%). We also included in the comparisons in Figure 3 the top model from the conventional LBA hierarchy. This model is more complicated (25 parameters) than either of the comparison models, and clearly it is able to accurately capture the correct RT distribution. However, it captures only 20% of the average speed-accuracy difference in error rates.

Figure 4 shows that the best LNR model and AIC-best LBA model captures the overall pattern in error RT in the speed-emphasis condition. In the accuracy emphasis condition there are fewer errors, particularly in for high-frequency words and non-words, so RT estimates are noisy. However, both models appear to systematically underestimate overall variability, resulting either in overestimation of the 10th percentile or underestimation of the 90th percentile. As was the case for correct RT, the simpler BIC-best LBA model, whose results are not shown, provides a similar account of error RT to the AIC-best LBA model.

**Figure 5:**
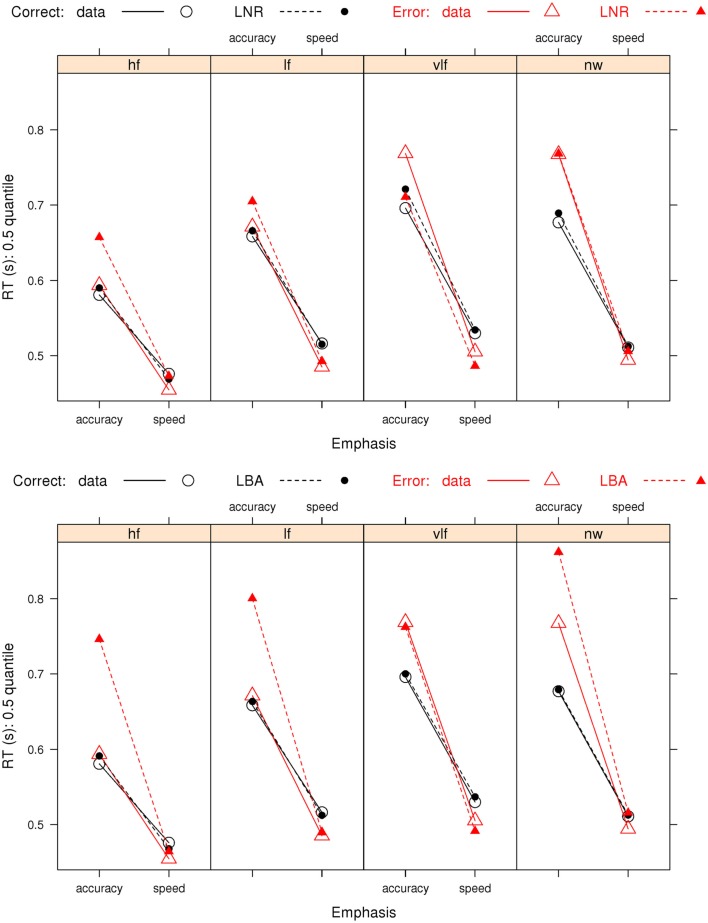
Observed and predicted median RTs for correct and error responses in Wagenmaker et al.’s (2008) Experiment 1 and fits averaged over participants for the best (selected by AIC and BIC) LNR μ ~ E*W*C & σ^2^ ~ E*C & *t*0* ~ E*) and best (selected by AIC) LBA (*B ~ *lR*E & *A ~ E* & v ~ W*C & sv ~ E*C & *t*0* ~ E*) models: hf, high-frequency words; lf, low-frequency words, vlf, very low-frequency words; nw, non-words.

Figure 5 focuses on the relative speeds of (median) correct and error RT, with confidence intervals omitted to make the pattern of results clearer. Both the best LNR and best LBA models capture the general pattern of slower error than correct responses under accuracy emphasis and faster error than correct responses under speed emphasis.

### Discussion

Our fits show that the LNR model is able to provide quite an accurate descriptive account of all of the effects in Wagenmaker et al.’s (2008) first experiment. Recall that the LNR model we fit is simplified in two senses; it assumes no variability in residual time, and it assumes inputs and distances are uncorrelated across accumulators. It is also without the LBA model’s freedom to combine trial-to-trial variability in distance with a distance greater than zero. That none of these restrictions caused a bad fit is not, by itself, evidence that these restrictions might not have to be relaxed in other situations. However, it does encourage the wider application of this simple and tractable form of the LNR model.

**Figure 6:**
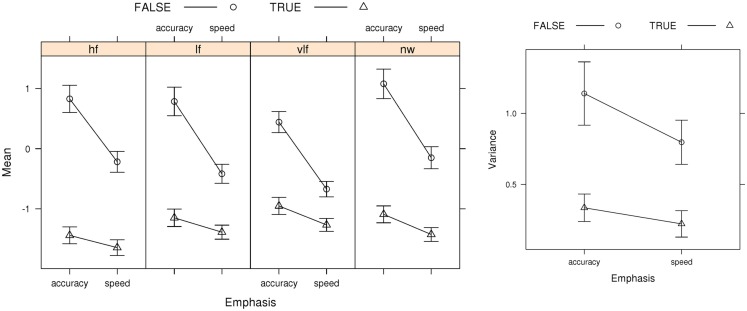
Best LNR (μ ~ E*W *C & σ^2^ ~ E*C & *t*0 ~ *E*) μ (Mean), and σ^2^ (Variance) parameter estimates averaged over participants, with bias-corrected within-subject 95% confidence intervals (Morey, 2008). FALSE, error-response accumulator; TRUE, correct-response accumulator; hf, high-frequency words; lf, low-frequency words; vlf, very low-frequency words; nw, non-words.

Figure 6 shows average mean (μ) and variance (σ^2^) parameter estimates for the best LNR model (i.e., the model selected by both AIC and BIC). The false accumulator (i.e., the accumulator corresponding to the error response) has a greater mean parameter than the true accumulator. Note that this results in an increase in both the mean and variance of boundary-crossing times for the false accumulator, because the μ parameter affects both. We also found that the false accumulator had a greater variance than the true accumulator, consistent with the example given in Figure 2. Again, this results in an increase in both the mean and variance of boundary-crossing times for the false accumulator, because the parameter σ parameter also affects both. These differences in μ and σmight arise, due to differences in the distributions of evidence accumulation rates for true and false accumulators. To provide a concrete example, suppose a template-matching process produces evidence for each response. If poorer matches produce outputs that at not only weaker on average but also more variable than outputs for strong matches the pattern displayed in Figure 6 could be found. In support of this possibility, there was a high positive correlation (*r* = 0.80, *p* < 0.001) between the mean and variance estimates for the top LNR model.

For both the LNR mean and variance, Figure 6 shows that the increase from speed to accuracy conditions is larger for false than true accumulators. This uneven increase cannot be explained by a selective influence of speed vs. accuracy emphasis on distance (i.e., the LNR analog of a boundary effect), as that must cause an equal effect on false and true accumulators (see Eq. 8). That is, accumulators are only “false” and “true” with respect to the stimulus, so the interactions evident in Figure 6 indicate that emphasis directly affects the mean and variability of the rate at which information is extracted from a stimulus. Clearly this conclusion is at odds with the way that speed-accuracy trade-off has traditionally been accounted for by evidence accumulation models. Note, however, that these results do not rule out some change in distance under accuracy emphasis, as long as this change is accompanied by appropriate changes in rates to yield the total effect in Figure 6.

**Figure 7:**
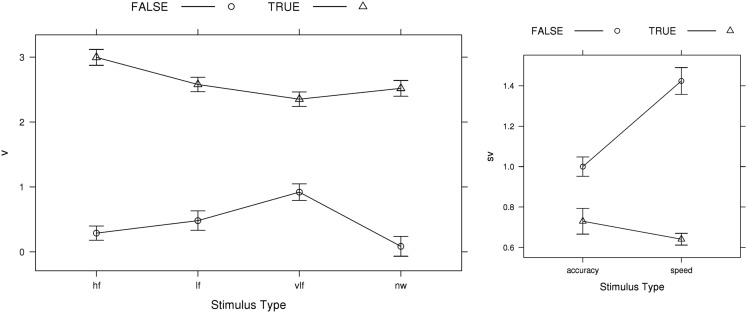
AIC-best LBA (*B ~ *lR*E & *A ~ E* & v ~ W*C & sv ~ E*C & *t*0* ~ E*) *v* (mean rate) and *sv* (rate standard deviation) parameter estimates averaged over participants, with bias-corrected within-subject 95% confidence intervals (Morey, 2008). FALSE, error-response accumulator; TRUE, correct-response accumulator; hf, high-frequency words; lf, low-frequency words; vlf, very low-frequency words; nw, non-words.

Perhaps more surprisingly, model selection failed to support the traditional boundary-only account for the LBA; the best model according to both AIC and BIC included emphasis effects on the LBA *sv* parameter. Figure 7 shows the rate-related parameter estimates for the AIC-best LBA model (BIC-best estimates were almost identical). The *sv* estimates were greater for the true than false accumulator, and vice versa for the *v* estimates, consistent with the findings for the analogous LNR parameters. In contrast to the LNR model, speed emphasis affected false accumulator variability in the opposite direction, increasing it greatly in the speed condition. Regardless, of this difference, clearly neither LBA nor LNR results are in line with conventional assumptions.

**Figure 8:**
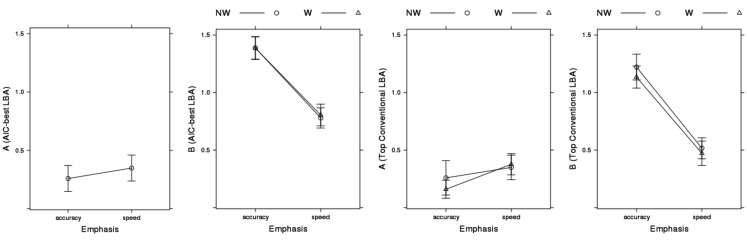
Emphasis effects on AIC-best (*B ~ *lR*E & *A ~ E* & v ~ W*C & sv ~ E*C & t0* ~ E*) and top conventional LBA model (*B ~ *lR*E & *A ~ *lR*E & v ~ W*C & sv ~ W*C & *t*0* ~ E*) start-point noise (*A*) and boundary (*B*) parameter estimates averaged over participants, with bias-corrected within-subject 95% confidence intervals (Morey, 2008). NW, non-word accumulator; W, word accumulator.

Our analysis of model fit indicated that effects of emphasis on the LBA boundary (*B*) and start-point noise (*A*) parameters, as well as on the *sv* parameter, were required to describe the large effects on error rate shown in Figure 3. Figure 8 plots these parameter estimates for the AIC-best LBA model. Speed emphasis caused a decrease in *B*, and to a lesser degree an increase in *A*. Figure 8 also plots results for the top conventional LBA model, which does not allow emphasis to affect any rate parameters. The pattern is similar, except that the effects are larger and the overall level of is *B* lower.

In summary, when free do to so, the LBA does not attribute the speed-accuracy trade-off induced by instructions in Wagenmaker et al.’s (2008) experiment purely to a change in response caution. Although changes affecting the level of errors caused by start-point noise (i.e., changes in the *A* and *B* parameters) could in principle accommodate the large observed differences in error rate, they are not able to do so while also providing an accurate account of RT distribution in this data. In particular, the strength of start-point noise in the LBA is limited because if it is too strong it can produce a more uniform RT distribution than is observed. Instead, the LBA, like the LNR, explains much of the speed-accuracy trade-off effect by an increase in the overlap of false and true distributions under speed emphasis.

## General Discussion

In this paper we have articulated framework for modeling simple-choice behavior using linear deterministic evidence accumulation. Within this framework the evidence is approximated as deterministic during accumulation, and the time for an evidence accumulator to reach its boundary is characterized by a ratio of two variables, one or more of which can vary randomly between-choice trials (“between-choice noise”). The numerator variable is the difference between the level of evidence at the start of accumulation and the boundary (“distance”) and the denominator variable is the rate of accumulation. Accumulators participate in a race that is non-interactive in the sense that that the state of one accumulator does not directly affect the state of other accumulators during accumulation. Response selection and RT are determined by the times at which evidence totals first cross one or more accumulator boundaries.

Different types of models within the linear deterministic framework correspond to different assumptions about the distributional forms of the variables in the ratio. Two previously proposed models that fall within the framework, Carpenter’s (1981) LATER model and Brown and Heathcote’s (2008) LBA model assume normally distributed rates; LATER also assumes distance is a constant whereas the LBA assumes it is uniformly distributed. Model types can also differ in three other ways: in how the random variables are related across accumulators, in the number of racers, and in how boundary crossings determine response selection. In the setup used in most previous applications of the LBA the random variables are uncorrelated, there is a one-to-one mapping of racers to responses, and the winning racer (i.e., the first to cross its boundary) triggers the corresponding response. However, it is also possible to have more racers than responses, to have responding contingent on more than one boundary crossing (see Eidels et al., 2010, for an LBA based example), and to have correlations among the random variables for different accumulators.

In this paper we proposed a new type of linear deterministic model, the LNR, and compared it to a slight variant of previous LBA models through fits of both models to Wagenmaker et al.’s (2008) speed-accuracy trade-off experiment. The version of the LNR that we focused on is mathematically simple because the random-variable ratio has the same distributional form as its constituents, a Lognormal distribution. A Lognormal ratio also results if the numerator (distance) is a constant and only the denominator (rate) is a Lognormal random variable or vice versa. The Lognormal form makes it tractable to allow rates and boundaries to be correlated over accumulators. However, in our initial exploration we assumed no correlation to see if this simple form of the LNR could still provide adequate fits. The LBA variant that we fit has a normal rate distribution truncated at zero, so on every trial all accumulators have a rate greater than zero. The latter property also applies to the LNR, as the Lognormal distribution is positive, so in both cases a response must eventually be selected on every choice trial.

The findings reported in this paper – both theoretical results related to the LNR and empirical results from fitting the LNR and LBA – bear most directly on the deterministic assumption made by our framework. On the theoretical front, the “flow” motivation of the LNR has implications for the division between stimulus encoding and response selection stages, and the effect of assuming a Lognormal distance has implications for the way in which deterministic models explain speed-accuracy trade-off by integrating out start-point noise. On the empirical front, our results point to the utility of the LNR as a tractable descriptive model and also highlight the issue of whether speed vs. accuracy emphasis instructions have effects beyond changes in the evidence boundary. Before addressing these implications, we first discuss the other fundamental assumption made by our framework, that accumulation is linear. As exemplified by the BA model (Brown and Heathcote, 2005a), there is no necessary relationship between the deterministic and linear assumptions, but linearity is closely related to another assumption, that accumulation is non-interactive.

### Linear accumulation

The nervous system is pervaded by non-linear dynamics, caused by factors such as imperfect (“leaky”) neural integration and recurrent self-excitation and lateral inhibition. Behavioral evidence for non-linear evidence accumulation has been sought using stimuli with non-stationary discriminative information, that is, stimuli that can briefly switch between the choices they support during the time course of accumulation. Usher and McClelland’s (2001) seminal experiments investigated the influence of the time within a stream of information favoring one response at which a brief pulse of contradictory information occurred. They found strong differences between participants ranging from leaky integration (i.e., a greater influence for late pulses occurring before response selection) through linear integration (sometimes abbreviated TSI for “time-shift invariance”) to results consistent with recurrent interactions (i.e., a greater influence of early pulses).

Huk and Shadlen (2005) performed a similar experiment on temporal integration of motion information in the lateral intraparietal (LIP) area of two rhesus monkeys. They found a greater influence for late pulses and concluded, based on modeling assuming within-choice noise, that: “… the time course of the pulse effects is consistent with the hypothesis that LIP reflects approximately linear integration that stops when the accumulation reaches a bound.” (p. 10443). Wong et al. (2007) simulated integration by a recurrent circuit perturbed by within-choice noise and found it displayed a: “violation of … TSI, similar to the violation observed in the Huk and Shadlen (2005) experiment.” (p. 8). Zhou et al. (2009) provide an insightful discussion of difficulties in distinguishing different types of integration using pulse paradigms.

Individual differences, and potential confounding of inferences about the nature of integration, might occur in these experiments if some participants noticed the pulse and employ compensatory strategies such as delaying the onset of sampling (to avoid being mislead by an early pulse) or prematurely terminating sampling (to avoid being mislead by a late pulse). Usher and McClelland (2001) attempted to minimize such problems using fairly brief pulses, as did Huk and Shadlen (2005) by making the pulse unrelated to rewards, but the success of these measures was not directly tested. Brown and Heathcote (2005b) attempted to avoid these problems using a very brief pulse (90 ms) that was meta-contrast masked. Masking was shown to be effective as detection of trials with a pulse was at chance levels for most participants. At the start of the experiment later arriving evidence had greater weight than earlier arriving evidence (i.e., accumulation was leaky), but as subjects practiced at the task integration quickly became linear.

One possible interpretation of why practice might promotes linear integration, is that it makes decision-making more efficient when evidence is stationary. That is, when early arriving evidence is no better guide to the correct choice than late-arriving evidence it is best to weigh each equally. In many simple-choice tasks where stimuli remain available until a choice is made, evidence is likely stationary, particularly as rapid responding means the stimulus is sampled for a relatively brief period of time. Stationarity does, however, require that sampling not begin prematurely (i.e., before stimulus information first becomes available), although Laming (1968) suggested the effects of premature sampling might be mimicked by start-point variability. When the stimulus is only available briefly evidence will be non-stationarity unless it is sampled from a mnemonic representation of the stimulus (e.g., Smith and Ratcliff, 2009). Of course, this type of non-stationary is different to the effects of non-linearity intrinsic to some evidence accumulation models, although they might mimic it.

More recently Tsetsos et al. (2011) introduced an innovative new multiple-pulse paradigm that manipulates temporal correlations among the evidence for three alternatives. In agreement with Usher and McClelland (2001), they found strong individual differences, and their analysis supported the LCA over two other within-choice noise models (a race and diffusion model). Overall, then, it appears that models that can accommodate all types of integration by adjusting the balance of leaky and recurrent dynamics best accommodate the full range of pulse-paradigm data. Such models include not only the LCA but also Wong et al.’s (2007) model, DFT (Busemeyer and Townsend, 1993), and Brown and Heathcote’s (2005a) BA. Even so, models assuming linear accumulation, such as the LBA and Ratcliff diffusion, often provide a good fit to data from the simple-choice paradigms used to evaluate evidence accumulation models. A potential reason is that large numbers of responses are collected in these paradigms in order to facilitate model fitting, and so participants are afforded substantial practice. Brown and Heathcote’s (2005b) results suggest that practice might cause an adjustment of recurrent interactions so they balance leakage, resulting in efficient integration that is approximately linear.

### Deterministic accumulation

That the deterministic assumption can support a comprehensive account of simple-choice behavior (i.e., of benchmark findings about the choices made and the times to make them) is perhaps surprising. The nervous system is not deterministic at the level of individual cell activity and most evidence accumulation models assume a dominant role for within-choice noise, although they can only provide a comprehensive account by also assuming a role for between-choice noise. The success of the deterministic assumption maybe less surprising, however, if it is kept in mind that it is an assertion about the importance of different types of noise for explaining choice responding. Importantly, this characterization as an approximation targeting behavioral measurement entails the implication that the deterministic assumption may not be appropriate for explaining other measurements of choice (e.g., single-cell firing rates). Even so, progress in science has often relied on developing approximations appropriate for different levels of description (e.g., the diffusive micro-scale behavior of gas molecules vs. the macro-level interactions between pressure, volume, and temperature captured by the gas laws).

The mechanism by which macro-scale deterministic behavior might emerge from the micro-scale variability intrinsic to the nervous system remains to be determined. Local averaging mechanisms have been argued not to be sufficient because of correlations between the activities of nearby cells that share inputs (Zohary et al., 1994). One possible explanation is that global rather than local brain dynamics determine behavior, and that it is these global dynamics emerging from the interaction of a variety of widely distributed areas that are deterministic. For example, Ho et al. (2009) suggested that activity consistent with evidence accumulation recorded in a variety of sensorimotor regions reflects a modality independent downstream input from right insula. Similarly, Forstmann et al. (2008) found that activity in the striatum and pre-SMA serves to implement the response boundary envisioned by evidence accumulation models. In both cases LBA model parameters provided a coherent link between observed behavior and neuroimaging measures.

Whatever the mechanism that achieves deterministic accumulation at the scale that determines behavior, it seems clear that deterministic accumulation could potentially give an organism a great adaptive advantage. Optimal methods of integrating out within-choice noise via accumulation have been of great interest on adaptive grounds (e.g., Gold and Shadlen, 2007). On these ground it might be even more desirable to remove noise intrinsic to the nervous system before accumulation (i.e., if accumulation was effectively deterministic), although in doing so there might be some trade-off with the level of between-choice noise. Regardless, given that no serious contender as a model of choice behavior can do without between-choice noise, it would seem to be desirable that optimality analyses take account of the effects of between-choice noise.

Our specific proposal that between-choice noise in the rate of linear accumulation has a Lognormal distribution has a novel implication for the conventional division between stimulus encoding and response selection stages in models of simple choice. This proposal can be derived from Ulrich and Miller’s (1993) idea that a cascade or “flow” of linear accumulation that characterizes all stages of processing from sensory processing up to and including response selection. A Lognormal distribution emerges as a Central Limit Theorem approximation when the rates of units in each stage are identically and independently distributed and vary independently between-choices, as assumed by Ulrich and Miller (1993), without strong assumptions on the form of that variation. If stimulus encoding does continuously feed activation into the response selection process, estimates of residual time might not, at least in part, reflect the time for stimulus encoding as is conventionally assumed. Instead, they would largely reflect the response execution stage, with only a small contribution from an initial “dead-time” before sensory neurons begin to respond.

Our mathematical results for an LNR with a Lognormal distance distribution might appear to show that not all linear deterministic models can explain speed-accuracy trade-off by integrating out random biases due to differences in the distance from starting point to boundary between accumulators. In the LBA the tendency for differences in rates to overcome random biases can be increased by an equal increase in the parameter controlling the position of the boundary (*b*) for all accumulators, resulting in slower but more accurate responses. An analogous effect is not obtained by increasing the LNR parameter determining the mean of the log-distance distribution, μ_D_, while holding the variance in log-distance, $\sigma_{\text{D}}^{\text{2}},$ constant. This is because and increase in μ_D_ increases not only the mean distance $\left(M_{\text{D}}={\text{e}}^{\mu_{D}+\sigma_{D}^{2}∕2}\right)$ but also the variability in distance $\left(V_{\text{D}}=\left({\text{e}}^{\sigma_{D}^{2}}-1\right){\text{e}}^{2\mu_{D}+\sigma_{D}^{2}}\right),$ and hence the magnitude of random bias. However, as pointed out by a reviewer, a change in *M*_D_ while holding *V*_D_ constant, although the effect is not exactly analogous to a change the *b*in the LBA (as it also changes the shape of the distance distribution), does result in a speed-accuracy trade-off in the LNR.

Future research could fruitfully explore not only different LNR parameterizations (such as in terms of *M*_D_ and *V*_D_) but also a wider variety of distributional assumptions. A range of positive distributions similar to the Lognormal, such as the Gamma distribution and extreme-value distributions such as the Weibull and Gumbel (see Heathcote et al., 2004), are plausible candidates for rate distributions. As they are positive, these distributions ensure that a response will be selected on every trial. Combinations of these rate distributions with different distance distributions (notably the analytically tractable shifted uniform distribution used in the LBA) will then help to provide a better understanding of the general strengths and limitations of the linear deterministic framework. A second area for further exploration concerns correlations in parameters across accumulators. The LNR model is particularly suited to such exploration, both because an LNR with such correlations remains tractable.

Our model fits show that the LNR can provide an accurate and comprehensive account of behavioral data from a simple-choice experiment. If this finding generalizes to other data sets the LNR could provide a useful descriptive model of simple-choice data. It is suited to this role because it sacrifices little of the tractability of distributions commonly applied to simple and correct-choice RTs (e.g., the Wald and ExGaussian, see Ratcliff and Murdock, 1976; Heathcote et al., 1991; Heathcote, 2004; Matzke and Wagenmakers, 2009), and is better able to describe differences in correct and error RT than the tractable EZ and EZ2 simplifications of the RDM (Wagenmakers et al., 2007; Grasman et al., 2009). Although both LBA and LNR likelihoods are easy to compute, the LNR has one distinct advantage: it is also easy to obtain full conditional likelihoods (i.e., likelihoods based on fixing all but one parameter). This is particularly useful for Bayesian approaches as it enables efficient Gibbs sampling steps rather than the less efficient methods required for the LBA (see Donkin et al., 2009a).

Our model fitting results could have the implication that, contrary to Brown and Heathcote’s (2005a, 2008) assertions, between-choice noise alone is insufficient to provide a comprehensive account of simple-choice behavior. This implication follows from the assumption that instruction manipulations cannot influence the rate of evidence accumulation. The LBA with this selective influence assumption has previously provided a comprehensive account of emphasis manipulations producing smaller effects on accuracy (e.g., Forstmann et al., 2008, 2010, 2011). In contrast, we found that this type of LBA was clearly unable to fit the changes in accuracy of up to almost 15% in Wagenmaker et al.’s (2008) data.

Although this selective influence assumption is conventional, recently there has been increasing evidence that instructions do influence drift rates, particularly in applications of the RDM. The RDM has always assumed that the relative values of the mean rates for stimuli associated with different responses are determined by a *drift criterion* parameter (Ratcliff and McKoon, 2008). Changes in the drift criterion and evidence boundaries both affect response bias but in different ways (e.g., Criss, 2010; Starns et al., 2012), and the drift criterion can be influenced by instructions (Leite and Ratcliff, 2011). Factors affecting attentional focus have also been argued to affect drift rates. For example, in White et al.’s (2011) RDM model of the flanker task drift rates change due to changes in “attentional focus” brought about by manipulating the proportion of trials involving response conflict. Arguably speed vs. accuracy emphasis instructions might also affect attentional focus, and hence the rate of information accumulation. Finally, Kleinsorge (2001) demonstrated that, given sufficient warning, participants can, in response to instructions, mobilize an extra effort that genuinely improves performance in a way that cannot be accounted for by a speed-accuracy trade-off. Given Wagenmakers et al. (2008) manipulated speed vs. accuracy instructions between blocks of trials participants had plenty of time to focus attention and/or make an extra effort that could affect performance through changes in drift rates.

On the basis of these recent findings, and the fact that the LBA and LNR did provide an accurate and comprehensive account of Wagenmaker et al.’s (2008) data when emphasis was allowed to affect drift rates, we believe it would be premature to reject the deterministic approximation. However, further research on this point is clearly called for. Neuroscience methods that can provide fairly direct evidence about the effect of instruction manipulations on the statistical characteristics (e.g., mean and variance) of evidence extracted from a stimulus will likely be particularly useful in this regard (see Ho et al., in press). Our results, and the others just reviewed, also suggest that it may also be fruitful to revisit traditional assumptions about the effects of a variety of instruction and expectancy (e.g., for a particular response or type of sensory information) manipulation in order to determine whether they can have multiple effects. That is, can these manipulations cause changes not only evidence accumulation parameters traditionally associated with strategic factors (e.g., boundaries and systematic bias) but also on rate means and on the variability of rates and biases?

## Conflict of Interest Statement

The authors declare that the research was conducted in the absence of any commercial or financial relationships that could be construed as a potential conflict of interest.

## Footnotes

^1^Density and cumulative density functions for the first “positive LBA” are obtained by dividing the corresponding expressions in Brown and Heathcote (2008) by Φ(*v*/*sv*). Heathcote and Hayes (2012) give details of the second, which introduces a dependency between rates for different accumulators.

^2^We limited the influence of outlying observations, which can be problematic for exact maximum-likelihood methods (Ratcliff and Tuerlinckx, 2002), by placing a floor on the likelihood of an observation corresponding to the assumption of a small (10^−5^) probability that participant’s data was contaminated by unbiased guesses with an RT drawn from a uniform distribution over the range from 0.3 to 2.5 s beyond which Wagenmakers et al. (2008) censored their data. We experimented with contamination probabilities up to 1% and obtained similar results.

^3^Models were not fit to these or any other set of percentiles; percentiles are only used to summarize goodness-of-fit. In contrast, the Ratliff diffusion model is usually fit to the 10th, 30th, 50th, 70th, and 90th percentiles, as that requires only calculation of the CDF, which is much quicker to compute for the RDM than the likelihood. We used exact maximum likelihood because this allows us to compute exact AIC and BIC values rather than relying on a multinomial-maximum-likelihood approximation (see Heathcote and Brown, 2004; Speckman and Rouder, 2004) required for percentile methods.
